# Activating adaptor-like sequences in pericentrin mediate its transport by dynein

**DOI:** 10.64898/2026.05.22.727342

**Published:** 2026-05-25

**Authors:** Wenzhu Zhang, Ami G. Sangster, Thao T. Nguyen, Samantha Melancon, Jia-Lin Shiu, Tian Hao Huang, Akhil G. Iragavarapu, Xueer Jiang, Jennielee Mia, Halil Aydin, Alan M. Moses, Li-En Jao

**Affiliations:** 1 Department of Biology, Syracuse University, Syracuse, New York, USA; 2 Department of Cell & Systems Biology, University of Toronto, Toronto, Ontario, Canada; 3 Department of Molecular Pathobiology, New York University, New York, New York, USA; 4 Department of Cell Biology and Human Anatomy, University of California, Davis, School of Medicine, Davis, California, USA

**Keywords:** Pericentrin, dynein, centrosome, pericentriolar material, coiled coils, co-translational transport, AlphaFold

## Abstract

Right before a cell divides, the centrosome rapidly increases its size and microtubule organizing activity through a process termed centrosome maturation. PCNT, a coiled-coil centrosomal protein, is synthesized and transported to the centrosome simultaneously by dynein. This dynein-mediated co-translational transport of PCNT facilitates centrosome maturation and mitotic spindle formation. How dynein engages and transports PCNT, however, remains unclear. Here we find that PCNT residues 1393–1525 are required for mediating dynein-dynactin interaction. PCNT (1393–1525) also shares similar sequence features with canonical dynein cargo adaptors. PCNT is predicted to interact with dynein heavy chains through similar contact sites used by canonical dynein cargo adaptors. Introducing point mutations at those contact sites abolishes dynein-mediated transport in a peroxisome motility assay. Our results suggest that PCNT contains adaptor-like sequences to bind and activate dynein directly during its transport to the centrosome. Our data also suggest that dynein may engage with some of its cargoes directly without an adaptor.

## Introduction

Centrosomes function as major microtubule organizing centers (MTOCs) in animal cells. Through nucleating and organizing microtubule arrays, they help regulate cell polarity, cell signaling, and intracellular transport (Conduit et al., 2015; Barlan and Gelfand, 2017). Centrosomes also facilitate the formation of the bipolar spindle to ensure faithful chromosome segregation during mitosis (Bettencourt-Dias and Glover, 2007; Bornens, 2012). The importance of centrosomes is further manifested by the plethora of human diseases linked to centrosome dysfunction, including cancers, brain disorders, dwarfism, and ciliopathies (Jaiswal and Singh, 2021).

The centrosome comprises a pair of centrioles surrounded by a proteinaceous network of pericentriolar material (PCM) (Rieder and Borisy, 1982; Vorobjev and Chentsov Yu, 1982; Wang et al., 2011; Woodruff et al., 2014; Conduit et al., 2015). The PCM contains hundreds of proteins (Andersen et al., 2003; Alves-Cruzeiro et al., 2014) and renders the MTOC activity of the centrosome (Gould and Borisy, 1977), as it harbors, among other proteins, the γ-tubulin ring complexes (γ-TuRCs) that nucleate microtubules (Moritz et al., 1995; Zheng et al., 1995; Jeng and Stearns, 1999; Oegema et al., 1999). In interphase, the centrosome has relatively small PCM in which many PCM proteins are organized as nanometer-sized toroids around the mother centriole (Fu and Glover, 2012; Lawo et al., 2012; Mennella et al., 2012; Sonnen et al., 2012). As the cell enters mitosis, however, the PCM expands dramatically into a micron-sized ensemble with a concomitant increase in microtubule nucleation activity in a process termed centrosome maturation (Khodjakov and Rieder, 1999; Mahen and Venkitaraman, 2012; Piehl et al., 2004; Palazzo et al., 2000; Mennella et al., 2014).

Pericentrin (PCNT) is a large coiled-coil protein (3,336 amino acids in humans) initially identified to be important for proper spindle organization (Doxsey et al., 1994). In vertebrates, a key function of PCNT is to initiate and recruit other PCM proteins during centrosome maturation (Zimmerman et al., 2004; Haren et al., 2009; Lee and Rhee, 2011; Lawo et al., 2012). PCNT and its orthologous proteins also organize interphase PCM. Regardless of cell cycle stages, the function of pericentrin in PCM assembly appears to be conserved among humans, mice, and flies, as loss of its activity results in the failed recruitment of the same set of downstream orthologous proteins in each system (e.g., CEP215 in human, Cep215 in mice, and Cnn in flies) (Lee and Rhee, 2011; Chen et al., 2014; Lerit et al., 2015). PCNT expression is also tightly regulated. Loss-of-function mutations in *PCNT* cause microcephalic osteodysplastic primordial dwarfism type II (Rauch et al., 2008; Griffith et al., 2008; Delaval and Doxsey, 2010), while elevated PCNT in Trisomy 21 disrupts ciliary protein trafficking, sonic hedgehog signaling, and may contribute to clinical features of Down syndrome (*PCNT* is located on Chromosome 21) (Galati et al., 2018).

Previously we show that human PCNT is recruited to centrosomes co-translationally during centrosome maturation (Sepulveda et al., 2018). By coupling translation and transport, this co-translational transport of PCNT is thought to promote timely production and incorporation of PCNT at mitotic centrosomes (Sepulveda et al., 2018). The middle segment of PCNT (residues 854–1960) can also undergo phase separation to form dynamic condensates, which also move toward centrosomes in interphase cells (Jiang et al., 2021). Both co-translational transport of endogenous PCNT and movement of PCNT (854–1960) condensates toward the centrosome are mediated by the dynein motor (specifically cytoplasmic dynein 1) (Sepulveda et al., 2018; Jiang et al., 2021). But how dynein engages and transports PCNT proteins/condensates along the microtubule remains unclear.

Dynein is a 1.4-MDa complex assembled from two copies of six polypeptides: the heavy chain (DHC), intermediate chain (DIC), light intermediate chain (DLIC), and three light chains (DLCs) (Trokter et al., 2012; Schmidt and Carter, 2016; Reck-Peterson et al., 2018; Canty et al., 2021). Mammalian dynein is autoinhibited and does not display processive motility (Trokter et al., 2012; Torisawa et al., 2014; Zhang et al., 2017). Activation of dynein requires the binding of its general cofactor dynactin (a 23-subunit, 1.2-MDa complex) and a cargo-specific adaptor protein (McKenney et al., 2014; Schlager et al., 2014; Carter et al., 2016; Reck-Peterson et al., 2018); only after this tripartite complex of dynein-dynactin-cargo adaptor is formed can the transport complex be activated and move processively along the microtubule with a specific cargo (McKenney et al., 2014; Schlager et al., 2014). Approximately 20 known dynein cargo adaptors have been identified to date. Despite limited sequence conservation (Reck-Peterson et al., 2018), they share two main sequence features critical for dynein-dynactin interactions and motor activation. The first is an N-terminal DLIC-binding (DLIC-B) domain, which folds differently among adaptor subfamilies (e.g., the CC1-Box, EF-hand, HOOK, or RH family) (Reck-Peterson et al., 2018; Olenick and Holzbaur, 2019; Singh et al., 2024). Despite using different folds, all DLIC-B domains form a hydrophobic cleft in which the same short helix at the C-terminus of DLIC is embedded (Lee et al., 2018, 2020). The second feature is a long coiled-coil region following the DLIC-B domain. It contains two general motifs—the HBS1 motif that binds the “tail” of DHC and the Spindly motif that interacts with the pointed-end subcomplex of dynactin (Urnavicius et al., 2015, 2018; Grotjahn et al., 2018; Chaaban and Carter, 2022; Singh et al., 2024). Specifically, the HBS1 motif, characterized by a weakly conserved glutamine residue followed by a patch of acidic residues, contacts the conserved Y^827^ and R^759^ residues of DHC, respectively (Chaaban and Carter, 2022; Singh et al., 2024; Aslan et al., 2026), while the Spindly motif, with a consensus sequence JxxEΦ for many, but not all adaptors (with J = leucine or isoleucine, Φ = any small hydrophobic) (d’Amico et al., 2026), binds p25 of the pointed-end subcomplex of dynactin (Gama et al., 2017; d’Amico et al., 2022; Chaaban and Carter, 2022; Singh et al., 2024; Aslan et al., 2026). Despite these sequence features found in all dynein cargo adaptors to date, the diverse DLIC-B domains among adaptor subfamilies, the lack of clear consensus sequence for the HBS1, and the short p25-binding Spindly motif make identify novel dynein cargo adaptors challenging by sequence homology search.

Early studies in cultured cells (Purohit et al., 1999; Tynan et al., 2000; Young et al., 2000) suggest that PCNT interacts with the middle region of DLIC (Tynan et al., 2000), a region different from where the DLIC-B domain of known dynein adaptors would bind. But whether this potential PCNT-DLIC interaction is direct and linked to dynein transport of PCNT remains unclear since the conclusion of these studies were drawn from co-immunoprecipitation of overexpressed PCNT and/or dynein subunits from cell lysates. The molecular mechanism underlying PCNT-dynein interactions and transport thus remains to be resolved.

In this study, we have used deletion analysis, live cell imaging, quantitative proteomics, evolutionary signature analysis (Pritišanac et al., 2026), AlphaFold structural prediction (Evans et al., 2021; Jumper et al., 2021), and *in cellulo* motility assays (Kapitein et al., 2010) to investigate how dynein engages and transports PCNT. We find that human PCNT residues 1393–1525 are required for mediating dynein interactions. Within PCNT (1393–1525), two HBS1-like motifs were predicted to interact with the DHC tails through the conserved Y^827^ and R^759^ residues. Reversing the charge of four acidic residues near the R^759^ contact abolishes the dynein-mediated transport in a peroxisome motility assay. Our results suggest that PCNT has built-in cargo adaptor-like sequences that engage with dynein-dynactin directly without a cargo adaptor.

## Results

### Human PCNT residues 1393–1525 are required for mediating PCNT-dynein interactions

**Figure 1.**
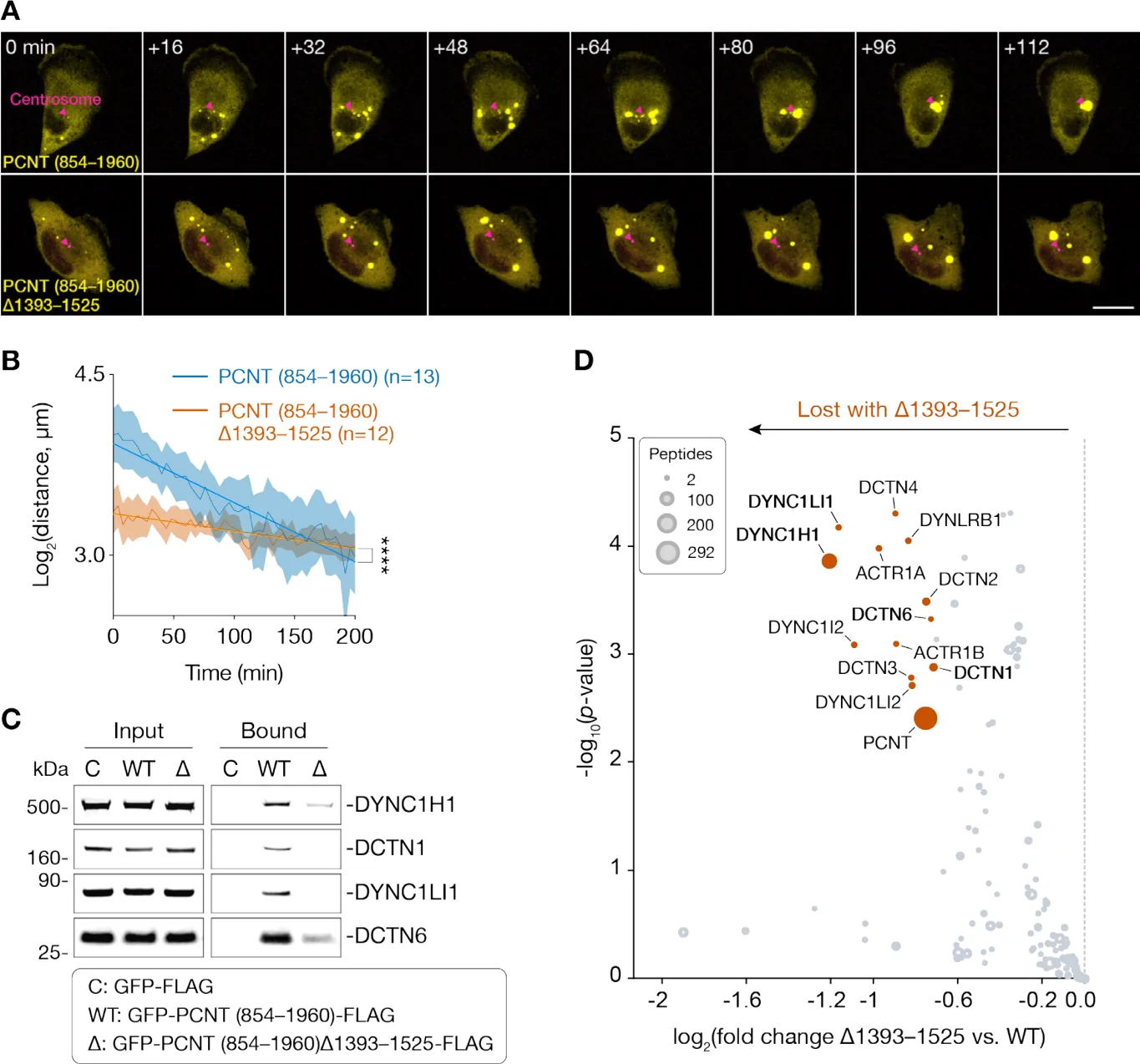
PCNT (1393–1525) is required for mediating the dynein transport and PCNT-dynein interactions. (**A**) Time-lapse micrographs of GFP-PCNT (854–1960) or GFP-PCNT (854–1960)Δ1393–1525 (yellow) expressed in RPE-1 cells stably expressing miRFP670nano3-CETN2 (magenta). Arrowheads denote the centrosomes. The time when the first condensates formed is marked as time 0. Similar results were obtained from more than three biological replicates. Scale bar, 20 μm. (**B**) Quantification of the distance of individual condensates to the centrosome over time. Data were aligned at the onset of phase separation (time 0) of individual condensates. Data are mean ± SEM. Statistical significance was determined by the *F*-test that compares the slopes of fitted lines between data sets via linear regression. **** *p*<0.0001. (**C**) Anti-GFP affinity purification of GFP-FLAG (C), GFP-PCNT(854–1960)-FLAG (WT), or GFP-PCNT(854–1960)Δ1393–1525-FLAG (Δ) was assayed by immunoblotting for the presence of dynein heavy chain (DYNC1H1), dynein light intermediate chain (DYNC1LI1), and dynactin subunits DCTN1 and DCTN6. Similar results were obtained from more than three biological replicates. (**D**) Six biological replicates of the immunoprecipitates described in (**C**) were analyzed by quantitative 18-plex tandem mass tag (TMT) mass spectrometry. Proteins de-enriched (⩾1.6-fold, *p*<0.005) were labeled (orange). DYNC1H1, DYNC1LI1, DCTN1, and DCTN6, the proteins assayed by immunoblotting (**C**), were among those de-enriched proteins (bold).

We previously showed that the middle segment of human PCNT, residues 854–1960 [i.e., PCNT (854–1960)], forms dynamic condensates that move toward the centrosome in a dynein-dependent manner in retinal pigment epithelial (RPE-1) cells (Jiang et al., 2021). To identify the sequences in PCNT (854–1960) responsible for mediating the dynein transport, we generated a series of GFP-tagged deletion variants of PCNT (854–1960) and tested the ability of the resulting condensates to move toward the centrosome by time-lapse microscopy. We found that the condensates formed by PCNT (854–1960) without residues 1393–1525 [i.e., PCNT (854–1960)Δ1393–1525] did not move toward the centrosome (Figures 1A, B).

To test whether PCNT (1393–1525) mediates PCNT-dynein interactions in cells, we first used GFP-tagged human BICD2 N-terminus (residues 25–423) (BICD2N) as a bait to purify dynein-dynactin complexes from RPE-1 cells by a nanobody-mediated purification strategy (Stevens et al., 2024) (Supplementary Figure S1). BICD2 is one of the most studied dynein cargo adaptors and BICD2N has been used to isolate native dynein-dynactin complexes from tissues or cells (e.g., McKenney et al., 2014; Okada et al., 2023). Immunoblot analysis showed that dynein heavy chain (DYNC1H1), dynein light intermediate chain (DYNC1LI1), and two dynactin subunits (DCTN1 and DCTN6) co-purified with BICD2N (Supplementary Figure S1C), validating our purification strategy in isolating native dynein-dynactin complexes from cells.

We next purified GFP, GFP-PCNT (854–1960), or GFP-PCNT (854–1960)Δ1393–1525 from RPE-1 cells using the same nanobody-mediated purification strategy. Immunoblotting demonstrated that a GFP-PCNT (854–1960) immunoprecipitate contained DYNC1H1, DYNC1LI1, DCTN1, and DCTN6, while GFP and GFP-PCNT(854–1960)Δ1393–1525 immunoprecipitates contained little to none of these proteins (Figure 1C). Quantitative mass spectrometry analysis of the immunoprecipitates further showed that various dynein and dynactin subunits were the most de-enriched proteins in GFP-PCNT(854–1960)Δ1393–1525 immunoprecipitates (Figure 1D). These results indicate that residues 1393–1525 of PCNT mediate PCNT-dynein interactions.

### PCNT (1393–1525) and known dynein cargo adaptors share similar molecular features

Dynein must bind its cofactor dynactin and a cargo adaptor (Reck-Peterson et al., 2018; Olenick and Holzbaur, 2019; Lee et al., 2020) to become active, capable of moving along the microtubule processively (McKenney et al., 2014; Schlager et al., 2014). Besides dynein and dynactin subunits, however, our quantitative mass spectrometry analysis failed to identify any known dynein cargo adaptors in the GFP-PCNT (854–1960) immunoprecipitate. This result suggests that PCNT binds dynein-dynactin through an unknown cargo adaptor or interacts with dynein-dynactin directly.

**Figure 2.**
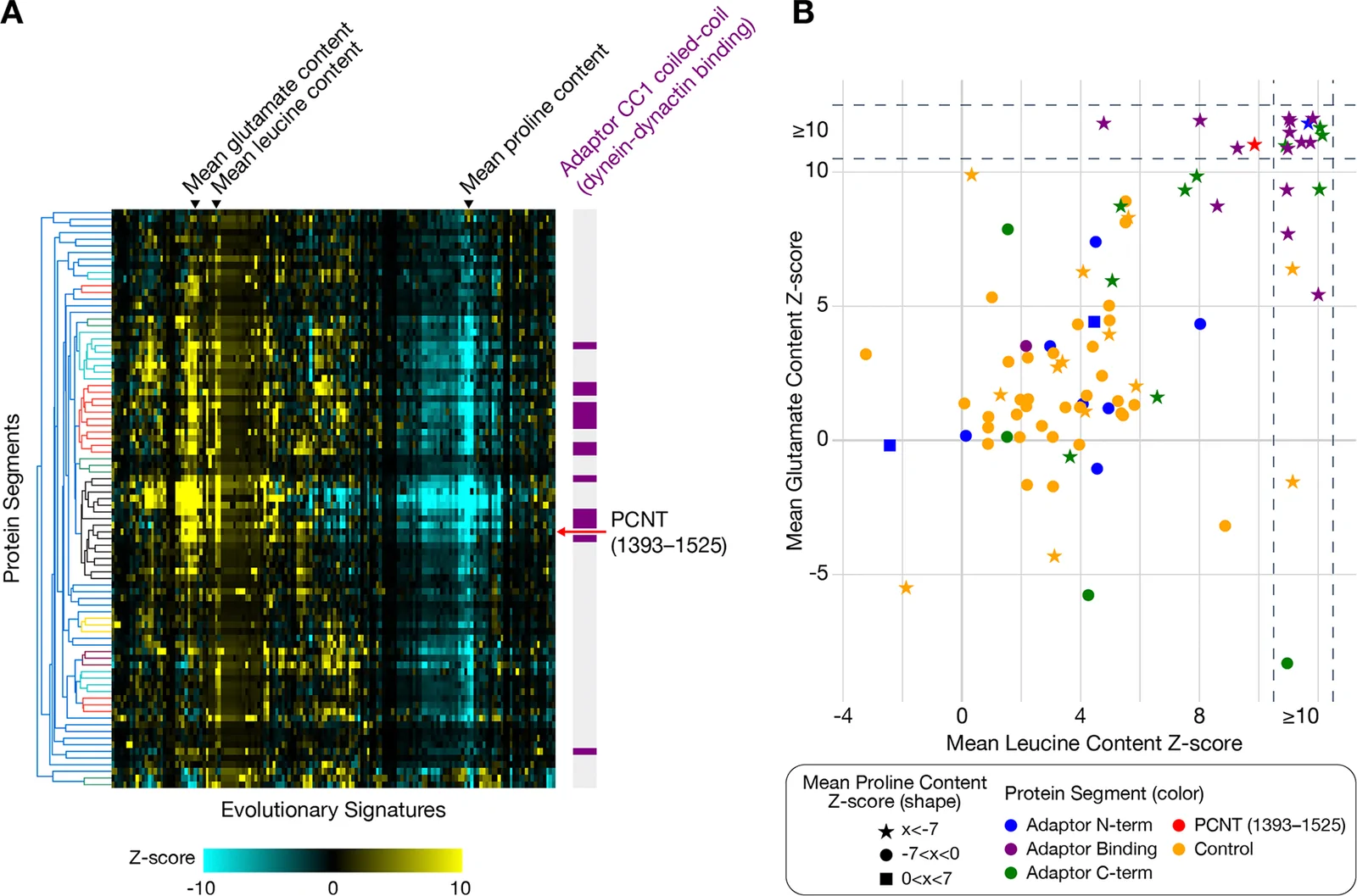
PCNT and dynein cargo adaptors share similar evolutionary signatures. (**A**) The evolutionary signature analysis of 87 protein segments, including segments of PCNT, dynein cargo adaptors, and annotated coiled-coils from UniProt. Each row corresponds to a protein segment and each column corresponds to the z-score of a sequence property measured in the evolutionary signature analysis. The heatmap includes a dendrogram to the left which indicates clusters defined by hierarchical agglomerative clustering at a threshold of 0.4. The bar graph to the right of the heatmap shows which rows contain the dynein-dynactin-binding segments (i.e., the CC1 coiled-coil) of the cargo adaptors (purple), and the row that corresponds to PCNT (1393–1525) (red arrow). (**B**) A scatter plot to show two of the highly conserved sequence features from the evolutionary signature analysis for the same 87 protein segments. Mean leucine content z-score is shown on the x-axis, mean glutamate content z-score is shown on the y-axis, mean proline context z-score is binned and shown as shapes, and the different types of protein segments are shown in different colors. Control refers to the set of annotated coiled-coils from UniProt. Z-score values are capped at ±10 and protein segments with values ⩾10 are shown between dotted lines across the top and right of the plot with random uniform noise added to separate the points.

Given that coiled-coil cargo adaptors share limited sequence conservation (Reck-Peterson et al., 2018) but all perform a shared function of activating dynein, we posited that cargo adaptors contain distinct molecular features that are under selection to preserve this shared function of interacting with dynein-dynactin. If PCNT interacts with dynein-dynactin directly, it should also share these distinct molecular features. To test this hypothesis, we used a computational pipeline (Pritišanac et al., 2026, also see Materials and Methods) to compute the evolutionary signatures of three groups of proteins: (1) sixteen known dynein cargo adaptors, each protein further divided into three segments: The B segment, which is the dynein-dynactin-binding long coiled-coil (i.e., the CC1 coiled-coil, between the DLIC-B domain and Spindly motif) (Hoogenraad, 2003; Lee et al., 2020; d’Amico et al., 2022; Canty et al., 2023; Singh et al., 2024; Teixeira et al., 2025; Aslan et al., 2026), and the N and C segments, which are the regions N-terminal and C-terminal to the B segment, respectively; (2) human PCNT, which is also divided into three segments as for the cargo adaptor, but the B segment here is PCNT (1393–1525) (i.e., the region mediating the PCNT-dynein interactions); (3) protein segments that correspond to any coiled-coil annotations from UniProt (The UniProt Consortium, 2025) that have a supporting publication to serve as negative controls (Supplementary Table S1). Figure 2 demonstrates that PCNT (1393–1525) shared similar evolutionary signatures with the B segments (i.e., the CC1 coiled-coil) of known dynein cargo adaptors, but not with the coiled-coils outside of the B segments nor with most of the annotated coiled-coil segments in UniProt. Among the highly conserved sequence features present in the adaptors’ B segments are the very high glutamate and leucine contents, but very low proline content (clustered at the upper-right corner of the scatter plot with star shapes in Figure 2B). PCNT (1393–1525) (red star) was also clustered at this region on the plot, while most of annotated coiled-coils (control, yellow) were not (Figure 2B). These results suggest that PCNT (1393–1525) and the CC1 coiled-coil of dynein cargo adaptors are under selection to preserve similar molecular features, implicating the role of PCNT (1393–1525) in mediating dynein-dynactin interactions in a manner similar to the cargo adaptors.

### PCNT (1393–1525) contains two putative HBS1 motifs

We next examined PCNT (1393–1525) for possible DLIC-B, HBS1, and Spindly motifs found in the cargo adaptors. We identified two HBS1-like motifs approximately 40–50 amino acids apart (we termed N-HBS1-like and C-HBS1-like for their relative N- and C-terminal positions) (Supplementary Figure S2). Each of these HBS1-like motif has one of the glutamine residues followed by a cluster of acidic residues, approximately 15 amino acids C-terminal to the glutamine (Supplementary Figure S2B). These glutamine and acidic residues are conserved in at least six vertebrates (Supplementary Figures S2C, D). This sequence architecture is similar to that of the HBS1 motif in several cargo adaptors (Supplementary Figure S2B) (Chaaban and Carter, 2022; Aslan et al., 2026; d’Amico et al., 2026). Structures from cryogenic electron microscopy (cryo-EM) and AlphaFold2 predictions have shown that the glutamine residue and the acidic patch in the HBS1 motif contact the conserved Y^827^ and R^759^ residues of DHC, respectively, in most cargo adaptors (Sacristan et al., 2018; d’Amico et al., 2022; Chaaban and Carter, 2022; Aslan et al., 2026; d’Amico et al., 2026).

### PCNT is predicted to interact with dynein heavy chains through HBS1-like motifs

**Figure 3.**
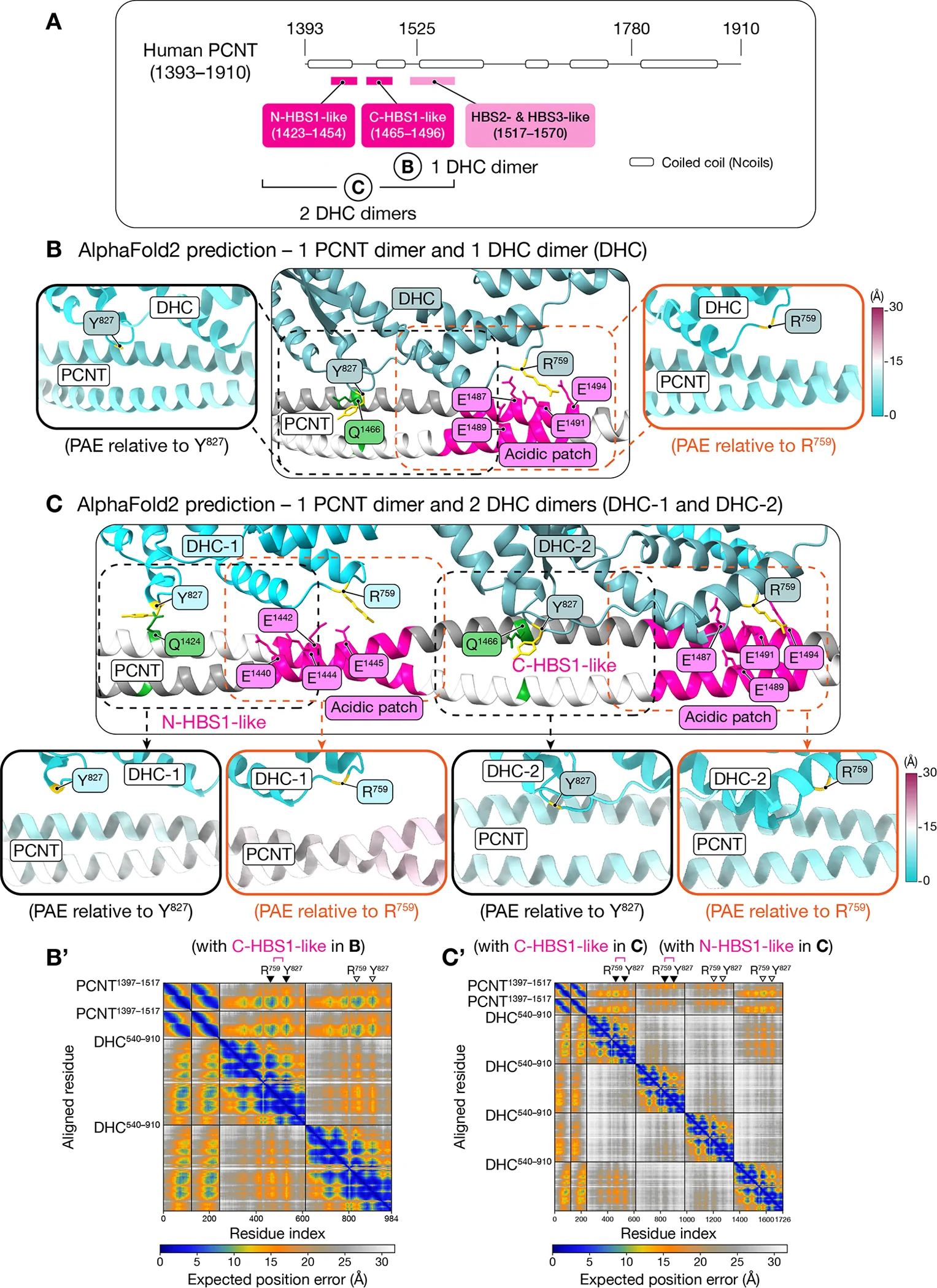
HBS1-like motifs of PCNT are predicted to interact with dynein heavy chains. (**A**) Domain structures of human PCNT (1393–1910) with the locations of HBS1-, HBS2-, and HBS3-like motifs (the ranges of amino acids in parentheses). Coiled-coil regions were predicted by Ncoils (Lupas et al., 1991). (**B**) AlphaFold2 models of one PCNT (1397–1517) dimer with one DHC (540–910) dimer. The zoom-in regions near Q^1466^ and the acidic patch in the C-HBS1-like motif are colored based on the predicted aligned error (PAE) values relative to the conserved DHC Y^827^ and R^759^ (yellow), respectively; lower values represent higher confidence. (**C**) AlphaFold2 models of one PCNT (1397–1517) dimer with two DHC (540–910) dimers. The zoom-in regions near Q^1424^ and Q^1466^ and the acidic patches in the N-HBS1- and C-HBS1-like motifs are colored based on PAE values relative to the conserved DHC Y^827^ and R^759^ (yellow), respectively. The full PAE plots are shown in **B’** and **C’** with solid arrowheads pointing to the positions of Y^827^ and R^759^ residues shown in the models in **B** and **C**.

We next used AlphaFold2 (Evans et al., 2021; Jumper et al., 2021) to determine whether PCNT might interact with DHC. Since dynein-dynactin-adaptor complexes can be formed with one of two dynein dimers (Urnavicius et al., 2015, 2018), we folded PCNT (1397–1517) with one or two DHC (540–910) dimers. Figure 3B shows that a PCNT dimer and a DHC dimer formed a complex in which the Q^1466^ residue and acidic patch of the C-HBS1-like motif were in close proximity to the conserved Y^827^ and R^759^ residues of DHC, respectively. However, when a PCNT dimer was folded with two DHC dimers (DHC-1 and DHC-2), both N-HBS1- and C-HBS1-like motifs engaged with the two DHC dimers individually, using the same architecture in which the glutamine residues (Q^1424^ and Q^1466^) and the acidic patches of PCNT interact with the Y^827^ and R^759^ residues of the two DHC dimers (Figure 3C).

### PCNT (1393–1910) acts like a canonical dynein cargo adaptor in cells

**Figure 4.**
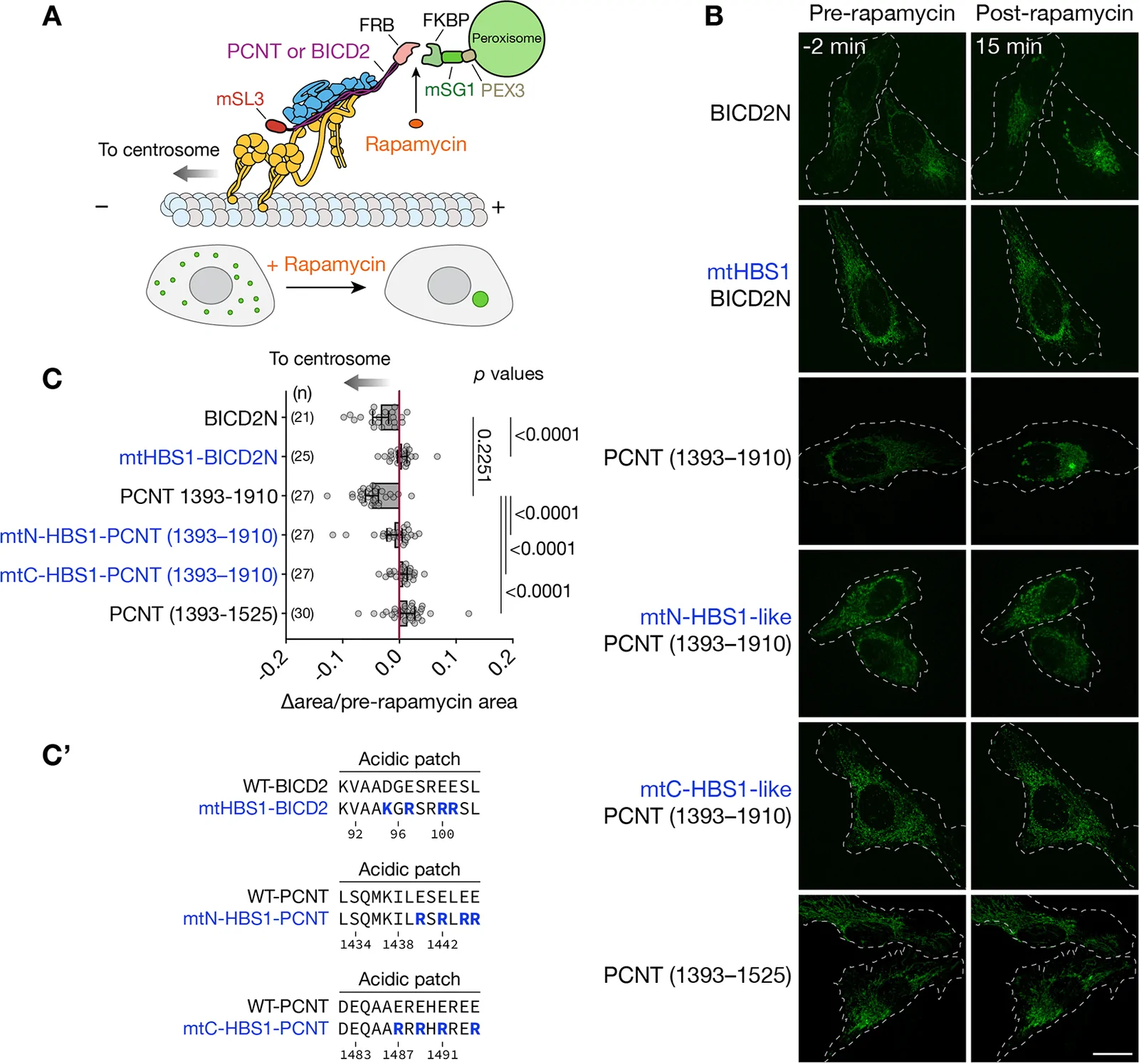
PCNT (1393–1910) acts like a canonical dynein cargo adaptor in cells. (**A**) Schematic of the inducible peroxisome transport assay (adapted from Aslan et al., 2025). HeLa cells were transiently transfected to express mScarlet3 (mSL3)-FRB fused BICD2 or PCNT variants, and peroxisome targeting sequence (PEX3)-mStayGold (mSG1) fused FKBP. Addition of rapamycin to the cell induces the localization of the dynein-dynactin-adaptor complex to peroxisomes and drives retrograde transport of peroxisomes toward the microtubule minus end. (**B**) Representative images of the peroxisome transport assay. Time 0 was the time when rapamycin was added. The cell was first imaged 2 minutes before rapamycin addition and imaged again 15 minutes post rapamycin addition. Scale bar, 20 μm. (**C**) Quantification of the peroxisome motility. Each data point is the ratio of the change in mSG1-positive area (15-min post- minus pre-rapamycin addition) to the pre-rapamycin mSG1-positive area per cell. The peroxisomes were scored as trafficking if the ratio is negative and there is a significant reduction in pre- and post-rapamycin areas (a two-tailed paired *t*-test). Data are represented as mean ± 95% CI from ⩾3 biological replicates. *p* values were calculated using one-way ANOVA with Dunnett’s test in comparison to the BICD2N or PCNT (1393–1910) condition. (**C’**) Locations of the charge reversal mutations in the acidic patches of BICD2 HBS1 and PCNT HBS1-like motifs. See Supplementary Figure S2B for the full sequences of the motifs.

To test whether PCNT can activate dynein motility in cells, we used an inducible peroxisome transport assay (Hoogenraad, 2003; Kapitein et al., 2010) to exogenously express a peroxisome targeting sequence (Kapitein et al., 2010) fused with mStayGold (mSG1) (Ando et al., 2023) and FK506 binding protein (FKBP) (Huynh and Vale, 2017; Wang et al., 2019), and different BICD2N and PCNT sequences fused with mScarlet3 (mSL3) (Gadella et al., 2023) and FKBP12-rapamycin binding domain (FRB) (Figure 4A). Treatment of the cell with rapamycin would induce the heterodimerization of FKBP and FRB and bring the peroxisomes and BICD2N or PCNT-fused proteins together. If BICD2N or PCNT construct acted as a dynein activating adaptor, the resulting peroxisome complexes would be transported toward the centrosome.

We found that as expected, BICD2N efficiently mediated the transport of peroxisomes to the centrosome upon rapamycin addition (Figures 4B, C). While PCNT (1393–1525) was not able to induce peroxisome trafficking to the centrosome, PCNT (1393–1780) (Supplementary Figure S3D) and PCNT (1393–1910) (Figures 4B, C) could do so as efficiently as BICD2N. Consistent with our AlphaFold models (Figure 3C), reversing the charges of the critical residues in contact with DHC R^759^ in either N-HBS1- or C-HBS1-like motif (ESELEE-to-RSRLRR or EREHEREE-to-RRRHRRER) completely abolished peroxisome trafficking (Figures 4B, C, C’). As a control, reversing the charges in the BICD2 HBS1 motif also abolished peroxisome trafficking (Figure 4D). These results suggest that PCNT can bind two DHC dimers through its two HBS1-like motifs and act like a canonical dynein cargo adaptor.

## Discussion

Activation of mammalian dynein requires the formation of a tripartite complex that contains dynein, dynactin, and one of the cargo adaptors bound with the cargo (McKenney et al., 2014; Schlager et al., 2014). PCNT is one of dynein’s cargoes and is transported to the centrosome by dynein co-translationally (Sepulveda et al., 2018). To determine how PCNT engages with dynein, we have used a series of cellular, biochemical, and computational approaches, as well as AlphaFold structural predictions, to determine the molecular basis of PCNT-dynein interactions. We find that PCNT residues 1393–1525 are required for mediating dynein-dynactin interactions, have the sequence features similar to those of the CC1 coiled-coil (i.e., the region responsible for bridging the dynein-dynactin interaction) of known cargo adaptors. Structural models indicate that PCNT (1393–1525) engages with dynein heavy chains (DHCs) through the sequences similar to the HBS1 motifs found in all cargo adaptors. Although PCNT (1393–1525) is not active in a peroxisome motility assay, PCNT segments with additional coiled-coils after residue 1525, such as PCNT (1393–1910), are able to drive retrograde transport of peroxisomes as efficiently as the positive control (i.e., BICD2N, the N-terminus of the canonical dynein cargo adaptor BICD2). Together, these data suggest that PCNT has built-in adaptor-like sequences, capable of engaging with dynein-dynactin directly without an adaptor.

### PCNT (1393–1525) and the CC1 coiled-coil of dynein cargo adaptors are under similar selection pressure

We find that PCNT (1393–1525) shares similar evolutionary signatures with those of the CC1 coiled-coils of sixteen known cargo adaptors. They share conserved sequence features enriched in charged, acidic residues, especially glutamate, as well as leucine, but depleted of glycine, polar residues, and proline (Figure 2). In contrast, the regions N- or C-terminal to the CC1 coiled-coils of the adaptors have different evolutionary signatures. Both PCNT (1393–1525) and adaptors’ CC1 coiled-coils have high propensity to form coiled-coils. However, coil-coil forming propensity is not the only feature under selection in this region, because most coiled-coil sequences do not have these features (Figure 2). Our data instead suggest that the capability of engaging with and activating dynein-dynactin complex is the key function the nature is selecting these sequences for. The preserved function apparently requires coiled-coils with additional properties, such as high acidic and leucine residue contents, and few prolines. These bulk sequence features are maintained despite many residue changes in the primary amino acid sequences. This conservation at the level of molecular features, not at primary sequences, also suggests that the binding interface formed by dynein-dynactin—also predominately coiled-coils—must have a high degree of plasticity to accommodate various adaptors and possibly certain cargoes directly (e.g., PCNT) with diverse sequences. Despite their simple structural architecture, coiled-coils have been recognized as one of the most versatile and diverse protein folds in nature (Lupas et al., 2017; Burkhard et al., 2001). The bulk sequence features that alter the properties of coiled-coils may represent unappreciated means by which diverse structural folds of coiled-coils are generated to perform different functions in the cell.

### PCNT binds one or two DHC dimers through two HBS1-like motifs

In our structural models, PCNT engages DHC through two HBS1-like motifs (N-HBS1-like and C-HBS1-like) (Figure 3), rather than through a single HBS1 motif found in most cargo adaptors. Similar to other cargo adaptors (Urnavicius et al., 2015; Chaaban and Carter, 2022), a PCNT dimer can also engage with one or two DHC dimers. When there is only one DHC dimer, PCNT is predicted to complex with DHC through the C-HBS1-like motif. When there are two DHC dimers, both N-HBS1-like and C-HBS1-like motifs of the PCNT dimer are predicted to engage with these two DHC dimers separately. In either case, all the interactions between PCNT and DHC dimer(s) are through a Q residue (approximately 15 amino acids N-terminal to the acidic patch) and several glutamate residues in the acidic patch of either HBS1-like motif of PCNT, and the conserved Y^827^ and R^759^ residues of DHC, respectively. This architecture of interactions resembles that of interactions between the HBS1 motifs of many canonical cargo adaptors and DHCs (Sacristan et al., 2018; Chaaban and Carter, 2022; d’Amico et al., 2022; Canty et al., 2023). We also find that reversing the charges of four glutamate residues surrounding the conserved R^759^ of DHC in either HBS1-like motif completely abolishes the PCNT-mediated peroxisome movement to the centrosome (Figure 4), suggesting that the peroxisome transport observed in our assay is specifically from PCNT-dynein interactions, as opposed to through an indirect mechanism, and that HBS1-DHC binding interface relies on charged interactions and is relatively sensitive to the perturbation caused by charge reversal.

While this manuscript was in preparation, d’Amico et al. reported that the cargo adaptor HOOK3 also has two consecutive HBS1 motifs that interact with two different DHC dimers in the cryo-EM structures (d’Amico et al., 2026). Having two HBS1 motifs in HOOK3 is unique among seven other adaptors. The two HBS1 motifs of HOOK3 are separated by approximately 40–50 amino acids, a similar distance between the two HBS1-like motifs in PCNT (Supplementary Figure S2). d’Amico et al. also found that most adaptors further interact with DHC after HBS1 through additional charged interactions (they refer to these HBS2 and HBS3) (d’Amico et al., 2026). Typically HBS1, HBS2, and HBS3 are approximately 30 amino acids apart. Interestingly, we identified two additional acidic patches that are well conserved in six vertebrates and are also approximately 30–60 amino acids C-terminal to the second HBS1-like motif (C-HBS1-like motif) in PCNT (Supplementary Figure S2D). These observations suggest that PCNT and HOOK3 share similar domain structures for HBS1, HBS2, and HBS3 motifs. Whether this similarity in domain architectures indicates a similar mechanism of engagement with DHC should become clearer when the structures of PCNT-DHC complexes are solved.

### The dynein-dynactin binding sites after PCNT (1393–1525)

Although PCNT (1393–1525) has the critical HBS1-like motifs to engage with DHC, it alone is not sufficient to drive peroxisome transport in our motility assay. However, both PCNT (1393–1780) (Supplementary Figure S3D) and PCNT (1393–1910) (Figure 4) are able to move peroxisomes to the centrosome efficiently. As in other adaptors, a Spindly motif after residue 1525 is likely present to support the formation of a stable PCNT-dynein-dynactin complex through the interaction with the pointed end of dynactin. However, we have not identified the Spindly motif through mutational analysis of several putative candidates by substituting the critical leucine and glutamate residues at positions 1, 4, and 5 for alanine residues, even though similar 3A mutations in the Spindly motif of BICD2N abolished the peroxisome transport (Supplementary Figure S3). The most promising Spindly motif candidate (residues 1771–1775) is near a break between the predicted coiled-coils, a key feature found in many Spindly motifs (d’Amico et al., 2022; Chaaban and Carter, 2022; d’Amico et al., 2026). However, introducing 3A mutations in this site failed to disrupt peroxisome trafficking (Supplementary Figure S3). Two possibilities may explain the results: (1) We simply have not identified and mutated the right Spindly motif. Atypical Spindly-like motifs divergent from the common consensus sequence have been reported (Aslan et al., 2026; d’Amico et al., 2026); PCNT might have an atypical Spindly motif not easily identifiable by sequence alone. (2) We mutated the correct Spindly motif, but its role in the peroxisome motility assay is dispensable. It has been shown that an interaction with the pointed end of dynactin is not absolutely required for some adaptor function. For example, deleting the Spindly motif in JIP3 has minimal effects on dynein motility *in vitro* (Singh et al., 2024). Besides Site 4 with p25 that interacts with the Spindly motif, the pointed end of dynactin also has additional sites for adaptor binding (e.g., through Site 2 and Site 3 on p62 and p25) (Lau et al., 2021). It is thus possible that disrupting the Spindly motif alone might disrupt dynein motility mediated by some (e.g., BICD2, Supplementary Figure S3), but not other adaptors, due to different degrees of contribution from those additional contact sites in adaptor/cargo binding.

In addition, d’Amico et al. also reported that two HOOK3 dimers interact with the dynein-dynactin complex in the cryo-EM structures (d’Amico et al., 2026). If PCNT resembles HOOK3 for not just having two HBS1 motifs, but also using two dimers to interact with dynein-dynactin at multiple binding sites, disrupting the Spindly motif alone might not abolish the PCNT-dynein-dynactin interaction. Making additional mutations at the potential interacting sites around the pointed end and solving the structures of PCNT with the pointed-end complex will help resolve these possibilities.

### The dynein-dynactin binding sites before PCNT (1393–1525)

It is not obvious that any known LIC1-B domain (e.g., CC1-Box, HOOK, EF-hands) is present N-terminal to the HBS1-like motifs in PCNT (1393–1910), but PCNT (1393–1780) or PCNT (1393–1910) can move peroxisomes toward the centrosome as efficiently as BICD2N does, which contains the LIC1-B domain CC1-Box. It is currently unknown whether an atypical LIC1-B domain is present at the N-terminal portion of PCNT (1393–1910) before HBS1-like motifs or whether PCNT (1393–1910) can bind and activate dynein-dynactin without binding to LIC1. It has been shown that albeit to a lesser extent, an adaptor (e.g., NINL, BICD2) without the LIC1-B domain can still activate dynein motility *in vitro* (Siva et al., 2025), suggesting that PCNT might also be able to activate dynein without the LIC1-B domain.

### Why do PCNT and some adaptors engage with dynein co-translationally?

Dynein moves PCNT toward the centrosome co-translationally (Sepulveda et al., 2018; Safieddine et al., 2021). Intriguingly, besides PCNT, at least three bona fide dynein cargo adaptors (NUMA1, NIN, and BICD2) and one putative adaptor (CCDC88C) (Redwine et al., 2017) are also transported to the centrosome co-translationally (Chouaib et al., 2020; Safieddine et al., 2021), presumably also through their engagement with dynein. Besides PCNT as a PCM scaffolding protein, all these four proteins function at the centrosome: NUMA1 focuses the microtubule minus ends (Merdes et al., 1996; Heald et al., 1996; Merdes et al., 2000; Sikirzhytski et al., 2014; Elting et al., 2014; Hueschen et al., 2017; Aslan et al., 2026), BICD2 helps tether the centrosome and the nucleus during G2 and M phases (Splinter et al., 2010; Gallisà-Suñé et al., 2023), NIN nucleates and anchors microtubules (Casenghi et al., 2005; Delgehyr et al., 2005; Redwine et al., 2017), and CCDC88C (also known as Daple) coordinates microtubule dynamics and ciliary positioning (Takagishi et al., 2017; Siletti et al., 2017). As the dynein transport occurs during active translation, dynein is expected to engage with the coiled-coil dimer formed by the nascent polypeptides emerging from separate ribosomes. Targeting the nascent adaptors to their destinations co-translationally (e.g., the centrosome in this case) may ensure that the adaptor only interacts with its cargo at the right place and time before the C-terminally located cargo-binding domains are made. It is also possible that the adaptor dimerization prefers to occur co-translationally to ensure proper assembly. How active translation and dynein-mediated transport are coordinated so that both processes can proceed smoothly remains to be resolved.

### A new mechanism for dynein to engage with its cargoes

Biochemical and structural evidence has now converged to suggest that dynein cargo adaptors have divergent sequences, but engage with dynein and dynactin through a few common anchor points, while a high degree of local variations in the architecture exists (Lau et al., 2021; Singh et al., 2024; Aslan et al., 2025; Teixeira et al., 2025; d’Amico et al., 2026). The discovery that PCNT has adaptor-like sequences that may bind and activate dynein directly without a cargo adaptor challenges the prevailing paradigm that mammalian dynein must rely on cargo adaptors for cargo engagement. This finding also suggests another dimension of plasticity in how dynein engages with their diverse cargoes. It remains to be discovered what other cargoes may engage with dynein and dynactin directly and how the regulation of this direct dynein-cargo engagement resembles and differs from that of the canonical adaptor-mediated dynein-cargo interactions.

## Methods

### Constructs

Chimeric PCNT constructs with *piggyBac* transposon elements and doxycycline (Dox)-inducible promoter were generated by replacing all or part of the PCNT sequence in PB-TA-sfGFP-PCNT (854–1960) (Jiang et al., 2021) with synthetic DNA (Twist Bioscience, South San Francisco, CA, USA). In all cases, the PB-TA-sfGFP backbone was PCR-amplified and assembled with the synthetic DNA fragments by isothermal Gibson assembly (Gibson et al., 2009). Constitutive active adaptor construct expressing sfGFP-tagged human BICD2 (25–423) (i.e., BICD2N) was generated in the same way by replacing the PCNT sequence in PB-TA-sfGFP-PCNT (854–1960) with the synthetic DNA encoding human BICD2 (25–423) (McKenney et al., 2014; Okada et al., 2023), where the numbers in parentheses represent amino acid positions.

Constructs used for assaying rapamycin-induced relocalization of peroxisomes were generated as follows. A peroxisome targeting sequence, human PEX3 (1–42) (Kapitein et al., 2010), fused to mStayGold (mSG1) (Ando et al., 2023) and FK506-binding protein 12 (FKBP), as well as mScarlet3 (mSL3) (Gadella et al., 2023) fused to human BICD2 (25–423) and FK506-rapamycin binding (FRB) domain, were synthesized and cloned into a lentiviral targeting plasmid pLVX-EF1α-mCherry-N1 (631986, Takara Bio, Mountain View, CA, USA) without the mCherry portion between the EcoRI and NotI sites by Gibson assembly, resulting in pLVX-EF1α-PEX3 (1–42)-mSG1-FKBP and pLVX-EF1α-mSL3-BICD2 (25–423)-FRB, respectively. Additional mSL3-tagged and FRB-fused adaptor constructs were generated by replacing BICD2 (25–423) in pLVX-EF1α-mSL3-BICD2 (25–423)-FRB via Gibson assembly or via site-directed mutagenesis (E0554S, New England BioLabs, Ipswich, MA, USA).

### Cell culture

hTERT-immortalized retinal pigment epithelial (hTERT RPE-1) cells were maintained in Dulbecco’s modified Eagle medium/Ham’s F-12 50/50 Mix (DMEM/F-12) (10–092-CV, Corning, NY, USA) and HeLa cells (ATCC CCL-2) were maintained in DMEM (10–017-CV, Corning). All cell lines were supplemented with 10% fetal bovine serum (FBS) (F0926, lot no. 21E578, MilliporeSigma, Burlington, MA, USA) and 1x Penicillin-Streptomycin-L-Glutamine (30–009-Cl, Corning), and maintained in a humidified incubator with 5% CO_2_ at 37°C. Cell lines used in this study were not further authenticated after obtaining from the sources. All cell lines were tested negative for mycoplasma using a PCR-based test with the Universal Mycoplasma Detection Kit (30–1012K, ATCC, Manassas, VA, USA). None of the cell lines used in this study was included in the list of commonly misidentified cell lines maintained by the International Cell Line Authentication Committee.

### Affinity purification of dynein complexes

A *piggyBac* transposon system (Kim et al., 2016; Jiang et al., 2021) was first used to generate stable RPE-1 cell lines that express various sfGFP-tagged “bait” proteins under the control of a doxycycline (Dox)-inducible promoter. After Dox induction, sfGFP or sfGFP-tagged PCNT, BICD2 baits and their associated proteins were purified using a nanobody (Nb)-mediated affinity purification strategy following a procedure described previously (Stevens et al., 2024). Briefly, approximately 2 × 10^7^ cells from two 15-cm dishes at about 90% confluence were trypsinized, washed with phosphate-buffered saline (PBS) twice, and lysed by a Dounce homogenizer with a tight pestle (06–435A, Fisher Scientific, Waltham, MA, USA) in the Nb-lysis buffer [20 mM HEPES – KOH, pH 7.5, 150 mM KCH_3_COO, 2 mM MgSO_4_, 1 mM EGTA, 10% glycerol, 0.1 mM ATP, 0.01% glyco-diosgenin (GDN), 1 mM DTT, 1 mM PMSF, and 1x cOmplete protease-inhibitor cocktail (11873580001, MilliporeSigma)]. Approximately 800 μl Nb-lysis buffer was used per 0.1 g of cell pellets. The homogenates were then cleared by centrifugation at 35 000 *g* for 35 minutes at 4°C and incubated with magnetic streptavidin beads, equivalent to 75 μl of bead slurry coated with 37.5 μg of biotinylated anti-GFP Nb fusion protein, for 1 hour at 4°C. After incubation, the beads were washed with Nb-wash buffer (= Nb-lysis buffer containing 0.4% Triton X-100) (1.25 ml, five times) and Nb-lysis buffer (1.25 ml, once). The bound proteins were then eluted by incubating the washed beads in 75 μl of the Nb-elution buffer (50 mM HEPES – KOH, pH7.5, 200 mM NaCl, 2 mM Mg(CH_3_COO)_2_, 0.5 mM ATP, 0.01% GDN, 0.05% Triton X-100,1 mM DTT, 1 mM PMSF, and 280 nM SENP^EuB^ protease) for 20 minutes on ice. The eluates were subjected to silver staining, Western blotting, and/or mass spectrometry analysis.

### Immunoblotting

Samples were resolved by 4–12% Bolt^™^ Bis-Tris Plus polyacrylamide gels (NW04125BOX, Thermo Fisher Scientific) and transferred to a polyvinylidene difluoride (PVDF) membrane. After transfer, the PVDF membrane was cut horizontally at 125-kDa protein marker and blocked in Intercept^™^ Blocking Buffer (927–60001, LI-COR, Lincoln, Nebraska, USA) for 1 hour at room temperature. The top half was then incubated with the anti-DYNC1H1 antibody (1:2000 dilution, 12345–1-AP, Proteintech, Rosemont, IL, USA) and anti-p150 [Glued] antibody (1:500 dilution, 610474, BD Biosciences, San Jose, CA, USA), and the bottom half was incubated with the anti-Dynein Antibody, 74 kDa Intermediate chains antibody (1:2000 dilution, MAB1618, MilliporeSigma) in Intercept^™^ Blocking Buffer for 1 hour at room temperature. After primary antibody incubation, membranes were washed with Tris-buffered saline (20 mM Tris – HCl, pH 7.6, 150 mM NaCl) containing 0.1% Tween-20 (TBS-T) for 15 minutes once, followed by three additional washes of 5 minutes each. Membrane was then incubated for 1 hour at room temperature with IRDye^®^ secondary antibodies (1:30,000 dilution, LI-COR) in Intercept^™^ Blocking Buffer. Membranes were washed again with TBS-T as described above (one 15-minute wash followed by three 5-minute washes), dried, and imaged using the Odyssey CLx Infrared Imaging System (Model 9140, LI-COR).

### Mass spectrometry

#### Sample preparation.

A buffer exchange was carried out using a modified SP3 protocol (Hughes et al., 2019). Briefly, approximately 250 μg of Cytiva SpeedBead Magnetic Carboxylate Modified Particles (65152105050250 and 4515210505250, Cytiva, Marlborough, MA, USA), mixed at a 1:1 ratio, were added to each sample. 100% ethanol was added to each sample to achieve a final ethanol concentration of at least 50%. Samples were incubated with gentle shaking for 15 minutes. Samples were washed three times with 80% ethanol. Proteins were eluted from SP3 beads using 200 mM EPPS, pH 8.5 containing Lys-C (129–02541, Wako, Osaka, Japan). Samples were digested overnight at room temperature with vigorous shaking. The next morning trypsin (90305, Thermo Scientific) was added to each sample and further incubated for 6 hours at 37°C. Acetonitrile was added to each sample to achieve a final concentration of approximately 33%. Each sample was labeled, in the presence of SP3 beads, with approximately 62.5 μg of TMTPro reagents (a total of 18 TMT channels, A44520 and A52045, Thermo Scientific, Liverpool, NY, USA). Following confirmation of satisfactory labeling (>97%), excess TMT was quenched by addition of hydroxylamine to a final concentration of 0.3%. The full volume from each sample was pooled and acetonitrile was removed by vacuum centrifugation. The samples were acidified with formic acid, desalted by StageTip, eluted into autosampler inserts (6PSV9–03FIVPT, Thermo Scientific), dried in a speedvac, and reconstituted with 5% Acetonitrile, 5% formic acid for LC-MS/MS analysis.

#### Liquid chromatography and tandem mass spectrometry.

Data were collected on an Orbitrap Eclipse mass spectrometer coupled to a Proxeon NanoLC-1200 UHPLC (Thermo Fisher Scientific). The 100 μm capillary column was packed in-house with 35 cm of Accucore 150 resin (2.6 μm, 150Å; ThermoFisher Scientific). Data were acquired for 180 minutes per run. A FAIMS device was enabled during data collection and compensation voltages were set at −40V, −60V, and −80V (Schweppe et al., 2019). MS1 scans were collected in the Orbitrap (resolution: 60,000; scan range: 400–1600 Th; automatic gain control (AGC): 4×105; maximum ion injection time: automatic). MS2 scans were collected in the Orbitrap following higher-energy collision dissociation (HCD; resolution: 50,000; AGC: 250%; normalized collision energy: 36; isolation window: 0.5 Th; maximum ion injection time: 86 ms.

#### Data analysis.

Database searching included all entries from the human UniProt database (downloaded in May 2021). The database was concatenated with one composed of all protein sequences for that database in the reversed order (Elias and Gygi, 2007). Raw files were converted to mzXML, and monoisotopic peaks were re-assigned using Monocle (Rad et al., 2021). Searches were performed with Comet (Eng et al., 2013) using a 50-ppm precursor ion tolerance and fragment bin tolerance of 0.02. TMT-pro labels on lysine residues and peptide N-termini (+304.207 Da), as well as carbamidomethylation of cysteine residues (+57.021 Da) were set as static modifications, while oxidation of methionine residues (+15.995 Da) was set as a variable modification. Peptide-spectrum matches (PSMs) were adjusted to a 1% false discovery rate (FDR) using a linear discriminant after which proteins were assembled further to a final protein-level FDR of 1% analysis (Huttlin et al., 2010). TMT reporter ion intensities were measured using a 0.003 Da window around the theoretical m/z for each reporter ion. Proteins were quantified by summing reporter ion counts across all matching PSMs. More specifically, reporter ion intensities were adjusted to correct for the isotopic impurities of the different TMT reagents according to manufacturer specifications. Peptides were filtered to exclude those with a summed signal-to-noise (S/N) <180 across all TMT channels and <0.5 precursor isolation specificity. The S/N measurements of peptides assigned to each protein were summed (for a given protein). Proteins considered to be significantly enriched or depleted were those with ⩾1.64-fold changes and FDR <0.05 (using student’s *t*-test followed by Benjamini-Hochberg correction for multiple comparisons (Benjamini and Hochberg, 1995).

### Evolutionary signature analysis

The evolutionary signatures of proteins segments were analyzed according to our established pipeline (Pritišanac et al., 2026) using codes from https://github.com/IPritisanac/IDR_ES. Briefly, we first collected orthologs for our selected proteins using OMADB (Kaleb et al., 2019) with a 1:1 mapping, aligned each orthologous set of proteins using MAFFT (Katoh and Standley, 2013), and selected out the amino acid sequences of orthologs that align with the protein segments of interest as described in Pritišanac et al. (2026). These orthologous sets of protein segments were then used by the evolutionary signature codes to measure an evolutionary signature for each protein segment of interest. An evolutionary signature is the z-score between the distribution of a measured sequence feature for the orthologous set of protein segments and a set of 1000 sequences from simulated evolution across 150 sequence features (Pritišanac et al., 2026). We set the largest magnitude of a z-score to be 10 and we dropped the sequence features “aromatic_spacing_meanZ” and “omega_aromatic_meanZ” due to some sequences not having aromatic amino acids. Last, we performed a clustering analysis on the evolutionary signatures using a plotly clustergram (https://dash.plotly.com/dash-bio/clustergram). We clustered both the rows and columns using hierarchical agglomerative clustering with average linkage and cosine distance. The following protein segments were not included in the analysis because the sequences are too short or they have poor ortholog alignments: Q32MZ4 128–250, Q9HCU9 51–98, N-SPDL1 1–22, N-RAB45 1–20, O60239 97–145, N-JIP3 1–11, N-NIN 1–7, B-JIP3 12–386, C-CDR2L 444–465, C-RAB11FIP3 713–756, C-JIP3 387–1336, Q9UHQ4 166–233.

### Structural predictions

Structure predictions were performed using the ColabFold implementation (Mirdita et al., 2022) of AlphaFold2 (Evans et al., 2021; Jumper et al., 2021). Models were generated with ColabFold v1.6.1 using AlphaFold-Multimer v3 weights, with 12 recycles per model and four random seeds. The PCNT-DHC complex shown in Figure 3B was modeled with two copies of PCNT residues 1397–1517 (UniProt O95613) and two copies of DHC residues 540–910 (UniProt Q14204), while PCNT-2DHC complex shown in Figure 3C was modeled with two copies of PCNT residues 1397–1517 and four copies of DHC residues 540–910 using the same parameters. No structural templates were used. Predicted aligned error (PAE) values relative to DHC residues Y^827^ and R^759^ were mapped onto the predicted structures using a custom Python script adapted from PointPAE (Chaaban and Carter, 2022, ; https://github.com/sami-chaaban/PointPAE; https://doi.org/10.5281/zenodo.6792801). The script extracted PAE values for each reference residue along both axes of the PAE matrix, averaged them, and generated a ChimeraX attribute file for visualization. Structures were visualized in UCSF ChimeraX (Goddard et al., 2018; Pettersen et al., 2021; Meng et al., 2023).

### Microscopy

Microscopy was performed using a spinning disk confocal microscope system (Dragonfly 620SR, Andor Technology, Belfast, UK) with 63x/1.40 (magnification/numerical aperture) or 100x/1.40 HC PL APO objectives (Leica, Wetzlar, Germany), coupled with 1x, 1.5x, or 2x motorized magnification changer. Image acquisition was controlled by Fusion software (Andor Technology) and images were captured with an iXon Ultra 888 EMCCD or ZL41 Cell sCMOS camera (Andor Technology). Sometimes deconvolution of the images was also performed using the Fusion software (Andor Technology).

All live cell imaging was performed with cells seeded in 35-mm glass-bottom dishes (P35G-1.5–10-C, MatTek Corp., Ashland, MA, USA or D35C4–20-1.5-N, Cellvis, Mountain View, CA, USA) mounted in a humidified chamber supplied with 5% CO_2_ inside a wrap-around environmental incubator (Okolab, Pozzuoli, Italy) with temperature set at 37°C.

### Peroxisome motility assay

HeLa cells were seeded onto 35-mm glass-bottom dishes at 70% confluence and transfected with plasmids encoding PEX3-mSG1-FKBP and mSL3-BICD2N-FRB, mSL3-PCNT (1393–1910)-FRB, or one of the BICD2N or PCNT variants using TransIT^®^-LT1 transfection reagent (MIR 2304, Mirus Bio, Madison, WI, USA) following manufacturer’s protocol. 24 hours post transfection, cells were treated with DMSO or 0.5 μM rapamycin and immediately imaged with a 63x or 100x objective for up to 20 minutes using a spinning disk confocal microscope (Dragonfly 620SR, Andor Technology). Image quantification was performed on Fiji (Schindelin et al., 2012). Briefly, Z-stacks were collapsed using maximum intensity projection, and background was subtracted using the rolling-ball method with the sliding paraboloid option. For each cell, a region of interest (ROI) was drawn around the cell boundary, and the area covered by peroxisomes and the area of the entire cell were defined as the areas containing 90% of total fluorescent signals (mSG1- and mSL3-positive, respectively) using a custom ImageJ macro. The macro ranked pixels by intensity in descending order and identified the minimum number of brightest pixels required to account for 90% of the total integrated fluorescence. This value was expressed as a fraction of the total number of pixels in the ROI, yielding the 90% fluorescence area for each channel. For each cell at each time point, we calculated a peroxisome-to-total cell area (P/T) ratio by dividing the 90% fluorescence area of the mSG1 signal by the 90% fluorescence area of the mSL3 signal. Peroxisome redistribution was calculated as a peroxisome redistribution index (PRI), which is the fractional change in the P/T ratios before and after rapamycin addition. Specifically, the P/T ratio measured 2 minutes before rapamycin addition ($P/T_{\text{pre}}$) was used as the baseline and compared with the P/T ratio at 15 minutes after rapamycin addition ($P/T_{\text{post}}$).


$$PRI=\frac{P/T_{\text{post}}\;-\;P/T_{\text{pre}}}{P/T_{\text{pre}}}$$

Negative PRI values indicate clustering of peroxisomes relative to the adaptor distribution, whereas positive values indicate dispersion.

Statistical analyses were performed in Prism 10 (GraphPad, Boston MA, USA). Three tests were applied. First, to determine whether peroxisomes were trafficked in the cell, a two-tailed paired *t*-test was used to compare the P/T ratios before and after rapamycin addition. Second, to assess whether each construct produced a significant change in peroxisome distribution from baseline, a one-sample *t*-test against a hypothetical mean of zero was performed for each condition. Third, to compare mutant constructs to their respective controls, two one-way ANOVAs were performed, each followed by Dunnett’s multiple comparisons test: (1) within the BICD2 series, BICD2 mutants (mtHBS1-BICD2N, mtSPDLY-BICD2N) were compared to BICD2N; and (2) within the PCNT series, PCNT truncations or mutants (1393–1525, 1393–1780, mtN-HBS1, mtC-HBS1, and mtSPDLY) were compared to PCNT (1393–1910).

## Supplementary Material

Supplement 1
