# Supplementary material for: Activating adaptor-like sequences in pericentrin mediate its transport by dynein
Source: bioRxiv. 2026 May 25:2026.05.22.727342. Preprint. [Version 1] doi: 10.64898/2026.05.22.727342 (PMC13232285; doi:10.64898/2026.05.22.727342)
Supplement: Supplementary file 1 [file NIHPP2026.05.22.727342v1-supplement-1.pdf]

bioRxiv preprint doi: https://doi.org/10.64898/2026.05.22.727342; this version posted May 25, 2026. The copyright holder for this preprint (which was not certified by peer review) is the author/funder, who has granted bioRxiv a license to display the preprint in perpetuity. It is made available under a CC-BY 4.0 International license.

Supplementary Information

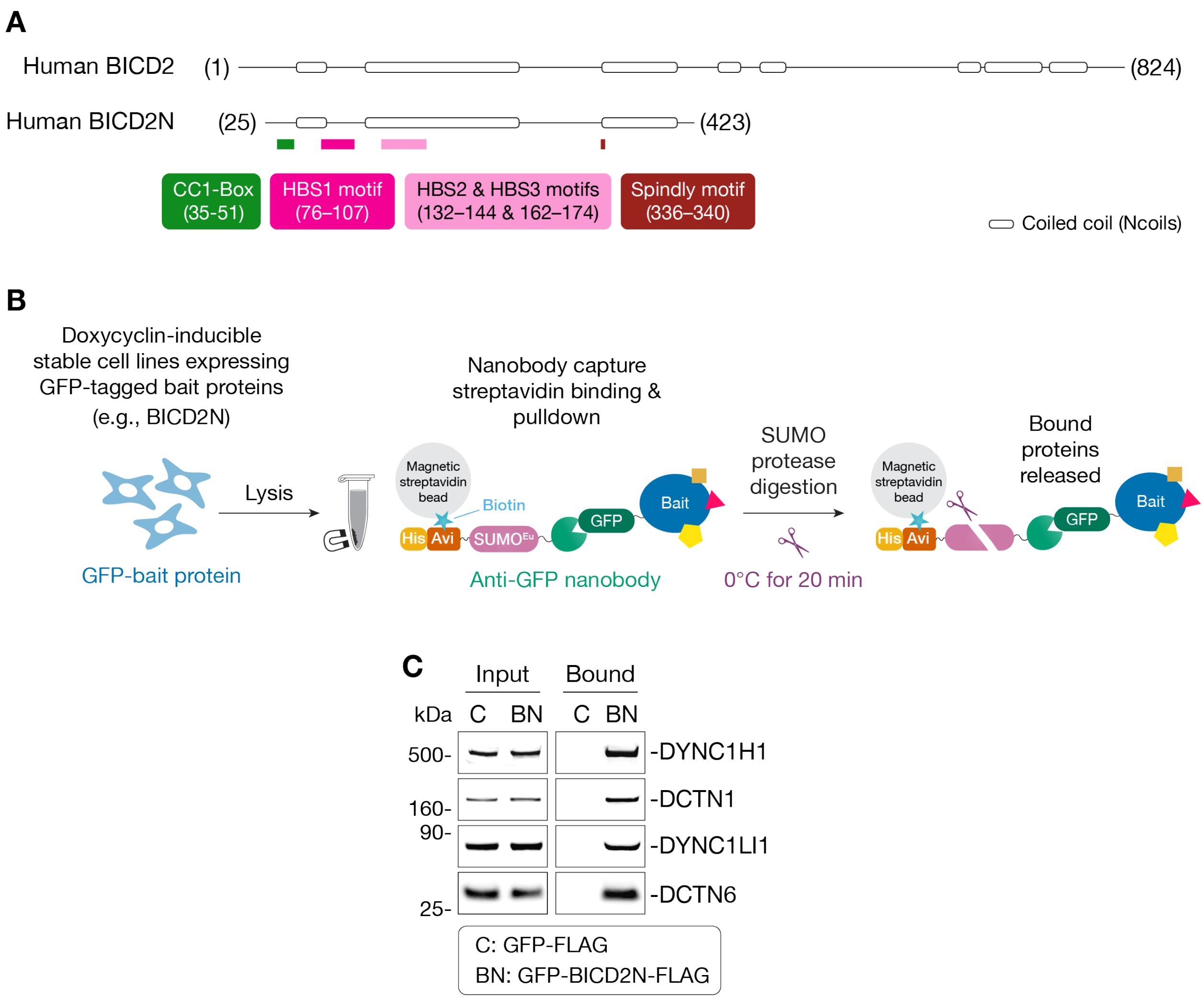

Figure S1. Nanobody-mediated affinity purification of dynein-dynactin-adaptor BICD2N complexes from RPE-1 cells. (**A**) Domain structures of human BICD2 (full-length and the N-terminal 25–423 residues). The locations of CC1-Box (DLIC binding), HBS1, HBS2, and HBS3 motifs (DHC binding), and Spindly motif (Dynactin pointed-end binding) are noted. The coiled-coil regions are predicted by Ncoils (Lupas et al., 1991). (**B**) Schematic of the anti-GFP nanobody-mediated purification protocol, a method adopted from Stevens et al. (2024). (**C**) Anti-GFP affinity purification of GFP-FLAG (C) or GFP-BICD2N-FLAG (BN) fusion protein was assayed by immunoblotting for the presence of dynein heavy chain (DYNC1H1), dynein light intermediate chain (DYNC1LI1), and dynactin subunits DCTN1 and DCTN6. Similar results were obtained from more than three biological replicates.

bioRxiv preprint doi: https://doi.org/10.64898/2026.05.22.727342; this version posted May 25, 2026. The copyright holder for this preprint (which was not certified by peer review) is the author/funder, who has granted bioRxiv a license to display the preprint in perpetuity. It is made available under a CC-BY 4.0 International license.

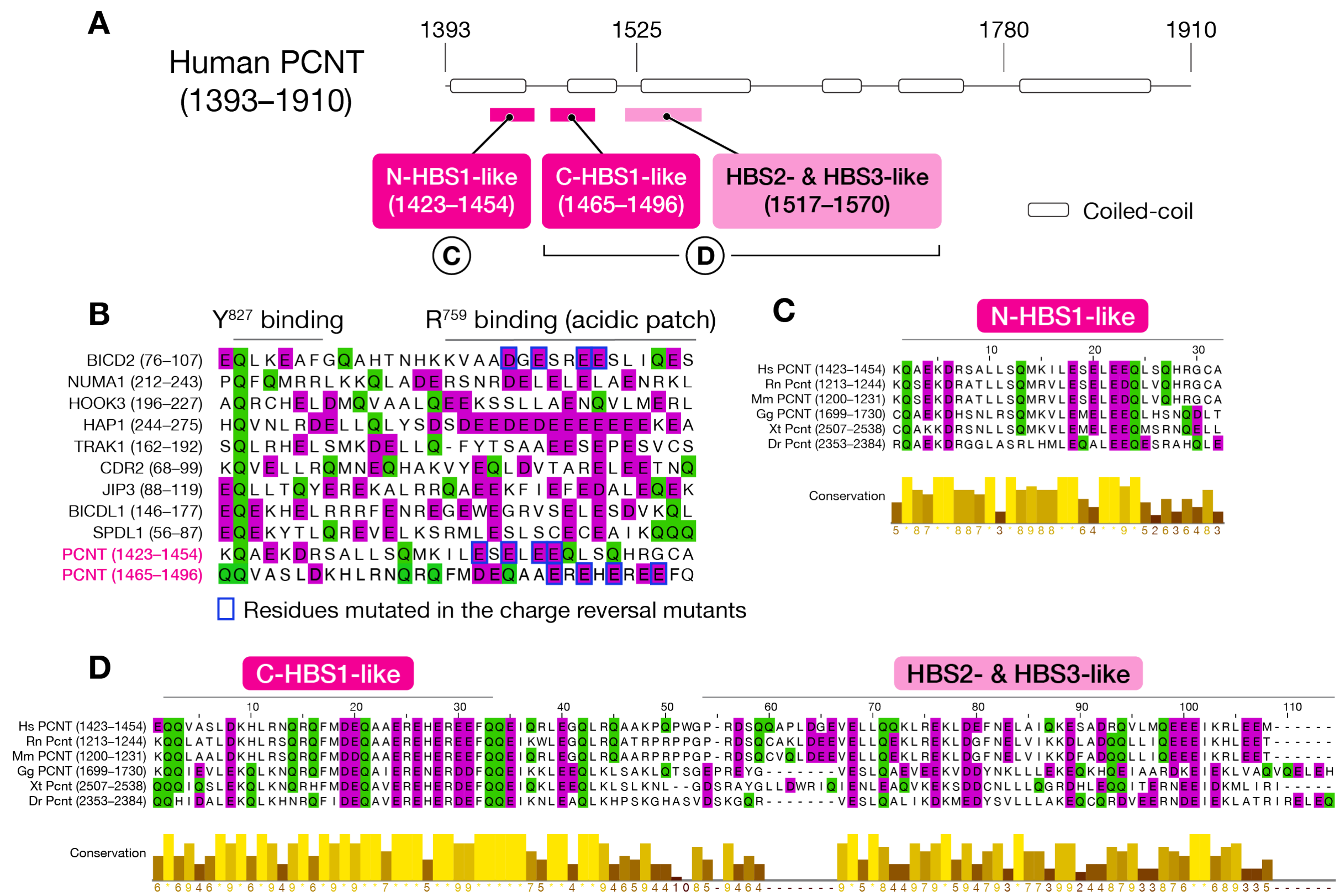

Figure S2. PCNT has HBS1-, HBS2- and HBS3-like motifs. (**A**) Domain structures of human PCNT (1393–1910) with the locations of HBS1-, HBS2-, and HBS3-like motifs (the ranges of amino acids in parentheses). Coiled-coil regions were predicted by Ncoils (Lupas et al., 1991). (**B**) Multiple sequence alignments of HBS1/HBS1-like motifs of nine dynein cargo adaptors and two HBS1-like motifs (i.e., N-HBS1- and C-HBS1-like) of PCNT. The approximate regions for interacting with DHC Y^{827} and R^{759} residues are noted. Blue rectangles mark the residues mutated in the charge reversal mutants of BICD2N and PCNT (1393–1910) analyzed in the peroxisome motility assay (**Figure 4**). (**C**) Multiple sequence alignments of N-HBS1-like motifs of six vertebrate PCNT orthologous proteins. (**D**) Multiple sequence alignments of C-HBS1-like, HBS2-, and HBS3-like motifs of six vertebrate PCNT orthologous proteins. Hs, *H. sapiens*; Rn, *R. norvegicus*; Mm, *M. musculus*; Gg, *G. gallus*; Xt, *X. tropicalis*; Dr, *D. rerio*.

bioRxiv preprint doi: https://doi.org/10.64898/2026.05.22.727342; this version posted May 25, 2026. The copyright holder for this preprint (which was not certified by peer review) is the author/funder, who has granted bioRxiv a license to display the preprint in perpetuity. It is made available under a CC-BY 4.0 International license.

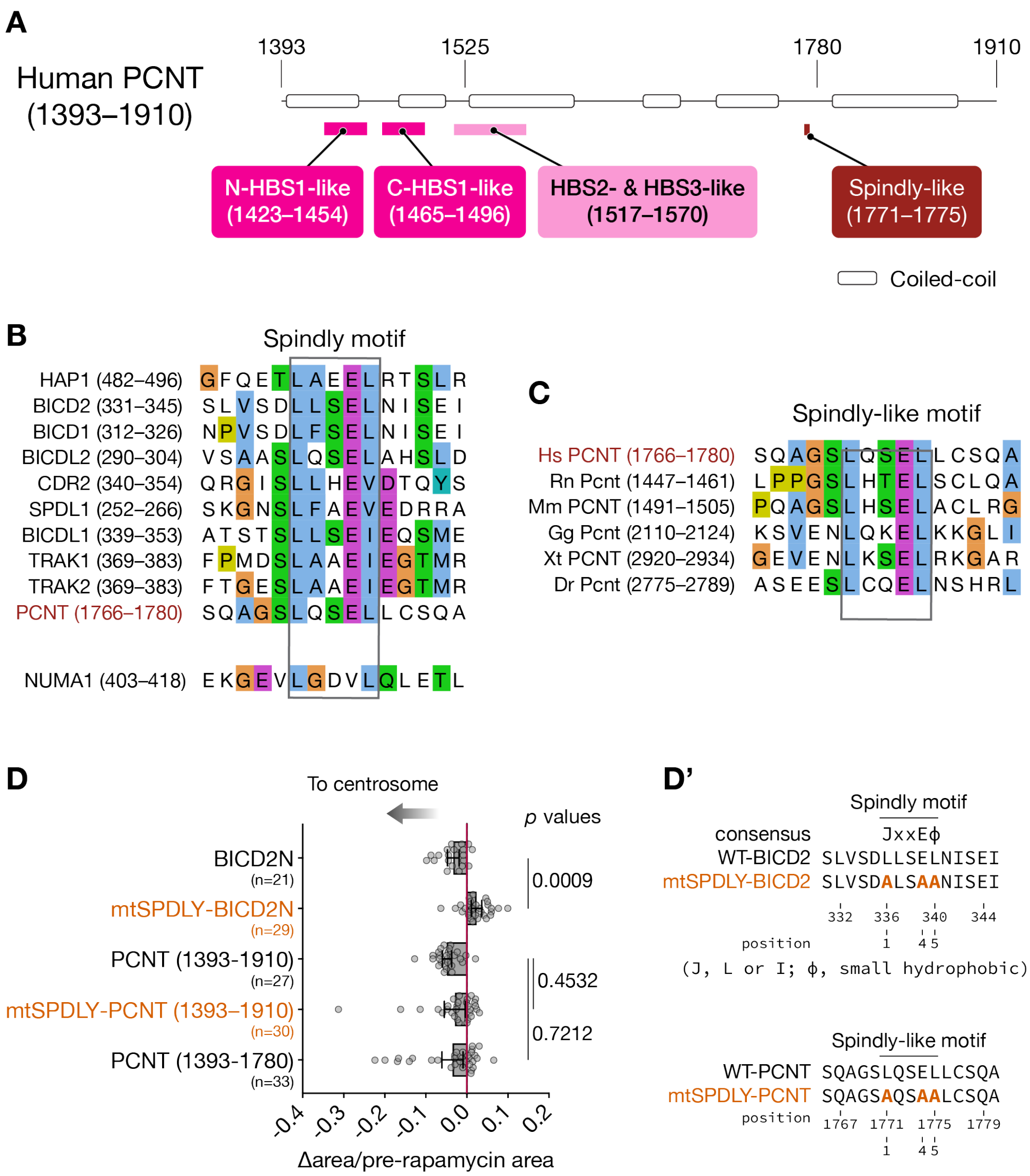

Figure S3. Effects of point mutations in the Spindly/Spindly-like motifs of BICD2 and PCNT (1391–1910) on peroxisome trafficking to the centrosome. (**A**) Domain structures of human PCNT (1393–1910) with the locations of HBS1-, HBS2-, and HBS3-like motifs, and one of the the putative Spindly-like motifs (the ranges of amino acids in parentheses). Coiled-coil regions were predicted by Ncoils (Lupas et al., 1991). (**B**) Multiple sequence alignments of Spindly/Spindly-like motifs of ten dynein cargo adaptors and PCNT. (**C**) Multiple sequence alignments of Spindly-like motifs of six vertebrate PCNT orthologous proteins. (**D**) Quantification of the peroxisome motility. Each data point is the ratio of the change in mSG1-positive area (15-min post- minus pre-rapamycin addition) to the pre-rapamycin mSG1-positive area per cell. The peroxisomes were scored as trafficking if the ratio is negative and there is a significant reduction in pre- and post-rapamycin areas (a two-tailed *t*-test). Data are represented as mean ± 95% CI from ⩾3 biological replicates. *p* values were calculated using one-way ANOVA in comparison to the BICD2N or PCNT (1393–1910) condition. (**D'**) Locations of alanine substitutions in positions 1, 4, and 5 of the Spindly/Spindly-like motifs of BICD2 and PCNT. Hs, *H. sapiens*; Rn, *R. norvegicus*; Mm, *M. musculus*; Gg, *G. gallus*; Xt, *X. tropicalis*; Dr, *D. rerio*.

bioRxiv preprint doi: https://doi.org/10.64898/2026.05.22.727342; this version posted May 25, 2026. The copyright holder for this preprint (which was not certified by peer review) is the author/funder, who has granted bioRxiv a license to display the preprint in perpetuity. It is made available under a CC-BY 4.0 International license.

Supplementary references

Lupas A, Van Dyke M, Stock J. Predicting Coiled Coils from Protein Sequences. Science (New York, NY). 1991; 252(5009):1162–4. doi: 10.1126/science.252.5009.1162.

Stevens TA, Tomaleri GP, Hazu M, Wei S, Nguyen VN, DeKalb C, Voorhees RM, Pleiner T. A Nanobody-Based Strategy for Rapid and Scalable Purification of Human Protein Complexes. Nature Protocols. 2024 Jan; 19(1):127–158. doi: 10.1038/s41596-023-00904-w.

bioRxiv preprint doi: https://doi.org/10.64898/2026.05.22.727342; this version posted May 25, 2026. The copyright holder for this preprint (which was not certified by peer review) is the author/funder, who has granted bioRxiv a license to display the preprint in perpetuity. It is made available under a CC-BY 4.0 International license.

Supplementary Tables

ID	GENE_ID	LENGTH	TYPE	EVIDENCE	POSITION
D6RGH6	MCIN_HUMAN	385	COILED	ECO:0000269|PubMed: 24064211	[178, 227]
O00161	SNP23_HUMAN	211	COILED	ECO:0000269|PubMed: 12556468	[22, 76]
O43715	TRIA1_HUMAN	76	COILED	ECO:0000269|PubMed: 26071602	[0, 52]
O60239	3BP5_HUMAN	455	COILED	ECO:0000269|PubMed: 30217979	[43, 90]
O60239	3BP5_HUMAN	455	COILED	ECO:0000269|PubMed: 30217979	[96, 145]
O60239	3BP5_HUMAN	455	COILED	ECO:0000269|PubMed: 30217979	[153, 200]
O60239	3BP5_HUMAN	455	COILED	ECO:0000269|PubMed: 30217979	[210, 255]
O60271	JIP4_HUMAN	1321	COILED	ECO:0000269|PubMed: 19644450	[407, 534]
O75030	MITF_HUMAN	526	COILED	ECO:0000305|PubMed: 24631970	[354, 402]
O75496	GEMI_HUMAN	209	COILED	ECO:0000269|PubMed: 15260975	[93, 144]
P02671	FIBA_HUMAN	866	COILED	ECO:0000305|PubMed: 19296670	[67, 631]
P02675	FIBB_HUMAN	491	COILED	ECO:0000305|PubMed: 10074346	[156, 222]
P08670	VIME_HUMAN	466	COILED	ECO:0000269|PubMed: 20176112	[95, 131]
P08670	VIME_HUMAN	466	COILED	ECO:0000269|PubMed: 20176112	[153, 245]
P08670	VIME_HUMAN	466	COILED	ECO:0000269|PubMed: 20176112	[302, 407]
P12883	MYH7_HUMAN	1935	COILED	ECO:0000255, ECO:0000269|PubMed: 26150528	[838, 1935]
P23246	SFPQ_HUMAN	707	COILED	ECO:0000255, ECO:0000269|PubMed: 25765647	[496, 596]
P35658	NU214_HUMAN	2090	COILED	ECO:0000269|PubMed: 19208808	[679, 1209]
P42224	STAT1_HUMAN	750	COILED	ECO:0000269|PubMed: 9630226	[135, 317]
P51572	BAP31_HUMAN	246	COILED	ECO:0000269|PubMed: 23967155	[164, 237]
P51787	KCNQ1_HUMAN	676	COILED	ECO:0000269|PubMed: 19693805	[584, 621]
Q09013	DMPK_HUMAN	629	COILED	ECO:0000269|PubMed: 15583383	[456, 536]
Q12933	TRAF2_HUMAN	501	COILED	ECO:0000269|PubMed: 20385093	[298, 348]
Q13563	PKD2_HUMAN	968	COILED	ECO:0000269|PubMed: 19556541	[832, 872]
Q13586	STIM1_HUMAN	685	COILED	ECO:0000269|PubMed: 22451904	[247, 442]
Q13614	MTMR2_HUMAN	643	COILED	ECO:0000269|PubMed: 16410353	[592, 627]
Q13976	KGP1_HUMAN	671	COILED	ECO:0000269|PubMed: 16131665	[1, 59]
Q15431	SYCP1_HUMAN	976	COILED	ECO:0000255, ECO:0000269|PubMed: 29915389	[738, 798]
Q32MZ4	LRRF1_HUMAN	808	COILED	ECO:0000269|PubMed: 23099021	[127, 250]
Q5HYA8	MKS3_HUMAN	995	COILED	ECO:0000269|PubMed: 34731008	[827, 917]
Q68DK7	MSL1_HUMAN	614	COILED	ECO:0000269|PubMed: 23084835	[212, 282]
Q8IZU3	SYCP3_HUMAN	236	COILED	ECO:0000255, ECO:0000269|PubMed: 24950965	[65, 223]
Q8N4S9	MALD2_HUMAN	558	COILED	ECO:0000269|PubMed: 28661558	[438, 548]
Q8ND30	LIPB2_HUMAN	876	COILED	ECO:0000269|PubMed: 21462929	[100, 313]
Q8WWY3	PRP31_HUMAN	499	COILED	ECO:0000269|PubMed: 17412961	[84, 120]
Q8WWY3	PRP31_HUMAN	499	COILED	ECO:0000269|PubMed: 17412961	[180, 215]
Q8WYA6	CTBL1_HUMAN	563	COILED	ECO:0000269|PubMed: 24269683	[475, 540]
Q96D96	HVCN1_HUMAN	273	COILED	ECO:0000269|PubMed: 20147290	[222, 266]
Q96RU3	FNBP1_HUMAN	617	COILED	ECO:0000269|PubMed: 17512409	[66, 259]
Q99623	PHB2_HUMAN	299	COILED	ECO:0000269|PubMed: 28017329	[189, 238]
Q99708	CTIP_HUMAN	897	COILED	ECO:0000269|PubMed: 34129781	[34, 84]
Q9H9Q4	NHEJ1_HUMAN	299	COILED	ECO:0000269|PubMed: 18046455	[127, 170]
Q9HC35	EMAL4_HUMAN	981	COILED	ECO:0000269|PubMed: 25740311	[13, 63]
Q9HCU9	BRMS1_HUMAN	246	COILED	ECO:0000269|PubMed: 21777593	[50, 98]
Q9HD67	MYO10_HUMAN	2058	COILED	ECO:0000269|PubMed: 23012428	[883, 934]
Q9P0L9	PK2L1_HUMAN	805	COILED	ECO:0000269|PubMed: 22193359	[699, 740]
Q9P270	SLAI2_HUMAN	581	COILED	ECO:0000269|PubMed: 21646404	[3, 39]
Q9UIL1	SCOC_HUMAN	159	COILED	ECO:0000269|PubMed: 24098481	[77, 146]
Q9ULV4	COR1C_HUMAN	474	COILED	ECO:0000269|PubMed: 12377779	[435, 474]
Q9UNL4	ING4_HUMAN	249	COILED	ECO:0000269|PubMed: 22334692	[24, 118]

Table S1. Coiled-coils from UniProt with supporting publications
